# Cognitive and behavioral performance in children with epilepsy with myoclonic–atonic seizures

**DOI:** 10.1590/1980-5764-DN-2024-0249

**Published:** 2025-07-11

**Authors:** Ericka Olívia Rodrigues Samoiloff, Denise Harumi Nakanishi, Eliane Correa Miotto

**Affiliations:** 1 Universidade de São Paulo, Faculdade de Medicina, Departamento de Neurologia, São Paulo SP, Brazil.; 2 Universidade de São Paulo, Faculdade de Medicina, Departamento de Neurologia, Disciplina de Neurologia Infantil, São Paulo SP, Brazil.

**Keywords:** Epilepsies, Myoclonic, Attention Deficit Disorder with Hyperactivity, Autistic Disorder, Neuropsychological Tests, Epilepsias Mioclônicas, Transtorno do Deficit de Atenção com Hiperatividade, Transtorno Autístico, Testes Neuropsicológicos

## Abstract

Epilepsy with myoclonic–atonic seizures (EMAS), or Doose syndrome, is characterized by the presence of atonic–myoclonic seizures that begin in childhood between 7 months and 6 years of age, which may present with cognitive and behavioral changes.

**Objective:**

The aim of this present study was to evaluate adaptive behavior, performance on intelligence and neuropsychological tests, and verify the association of autism spectrum disorder and attention deficit hyperactivity disorder in patients diagnosed with EMAS compared with a control group of healthy children.

**Methods:**

We included nine patients with EMAS and nine healthy controls, assessed by scales of adaptive behavior development, autism spectrum disorder, attention deficit and hyperactivity, and intelligence and neuropsychological tests.

**Results:**

The results revealed that in the intelligence and neuropsychological tests, there was a significant difference between the groups (p>0.05), with worse performance for the EMAS group. In the latter group, eight patients showed some symptoms of attention deficit hyperactivity disorder and none showed symptoms of autism spectrum disorder or changes in adaptive behavior.

**Conclusion:**

These findings show the relevance of investigating cognitive and behavioral profiles in this population in order to address specific impairments in their everyday life activities.

## RESUMO.

A epilepsia mioclônico-atônica (EMA) ou Síndrome de Doose, é caracterizada pela presença de convulsões mioclônico-atônicas de início na infância entre os 7 meses aos 6 anos de idade, que podem ocasionar alterações cognitivas e comportamentais.

**Objetivo:**

O presente estudo avaliou o comportamento adaptativo, o desempenho nos testes de inteligência, neuropsicológicos, e a associação do transtorno do espectro autista, transtorno do déficit de atenção e hiperatividade, nos pacientes com diagnóstico de EMA ou Síndrome de Doose, comparados com um grupo controle de crianças saudáveis.

**Métodos:**

Foram incluídos nove pacientes com EMA ou Síndrome de Doose e nove controles saudáveis, avaliados por escalas de desenvolvimento do comportamento adaptativo, transtorno do espectro autista, déficit de atenção e hiperatividade, testes de inteligência e neuropsicológicos.

**Resultados:**

Nos testes de inteligência e neuropsicológicos houve diferença significativa entre os grupos (p<0,05), com pior desempenho para os pacientes com EMA ou Síndrome de Doose. Nos testes de inteligência e nos testes neuropsicológicos houver diferença significativa entre os grupos (p>0,05), com pior desempenho para os pacientes com Síndrome de Doose. No grupo de EMA ou Doose oito pacientes apresentaram sintomas do transtorno de déficit de atenção e hiperatividade e nenhum apresentou sintomas estatisticamente significativos de transtorno do espectro autista ou de alteração no comportamento adaptativo.

**Conclusão:**

Esses achados mostram a relevância de se avaliar o perfil cognitivo e comportamental desta população para que prejuízos específicos que impactam nas atividades de vida diária sejam acompanhados.

## INTRODUCTION

Epilepsy has been defined as a brain dysfunction characterized by a predisposition to generate epileptic seizures. An epileptic seizure is a brief occurrence of signs and/or symptoms due to excessive or synchronous abnormal neuronal activity in the brain. The classification of epilepsy, established by the International League Against Epilepsy (ILAE), considers the type of seizure, the underlying etiology, the electroencephalographic pattern, and other clinical characteristics. Manifestations vary and may involve motor, sensory, autonomic, cognitive, and behavioral systems^1^.

Seizures can be focal or generalized. A generalized seizure is characterized by the involvement of both cerebral hemispheres from the onset, as evidenced by bilateral, symmetric, and synchronous epileptiform discharges on electroencephalography (EEG). The understanding of generalized seizures has evolved with advanced neuroimaging and electrophysiological studies, which suggest that while these seizures appear generalized, they may have focal cortical origins with significant thalamic involvement, highlighting the complexity of their network dynamics. Focal seizures originate in neural networks limited to one hemisphere^2-4^.

The EEG is a useful complement for classifying seizure type, especially in focal epilepsies in which epileptiform activity can help identify a lobar location.

In addition, cognitive and behavioral assessments are relevant tools to investigate the type and level of cognitive impairment and the presence of maladaptive behavior. By obtaining clinical, cognitive, and behavioral information about the patient, in addition to seizure types, age of onset, and associated comorbidities, it can contribute to the diagnosis of a specific epilepsy syndrome^2,5,6^.

Childhood epilepsies are complex and encompass idiopathic generalized epilepsies such as juvenile myoclonic epilepsy (JME), Dravet syndrome, Doose syndrome, West syndrome, epilepsy with focal migratory seizures, Lennox–Gastaut syndrome, Rett syndrome, and developmental and epileptic encephalopathies. However, further studies investigating cognitive and behavioral performance in this population are needed^7^.

In the study by Iqbal et al.^8^, executive function, intelligence, visuospatial skills, language, memory, attention, anxiety, depression, emotional, and behavioral traits related to executive dysfunction in patients with JME were investigated, comparing their performance with siblings and healthy controls under video EEG (video-EEG) conditions. Notably, 22 pairs of siblings, one of them with JME, matched for age, sex, and education, underwent neuropsychological testing and a self-report questionnaire. The JME group differed significantly from controls on measures of phonemic and semantic verbal fluency. They scored significantly higher on the dysexecutive self-report questionnaire, being more likely to report characteristics associated with executive dysfunction than siblings and controls. Patients significantly demonstrated worse outcomes relative to controls in psychomotor speed, phonemic verbal fluency, and characteristics associated with executive dysfunction. The results showed a tendency for the JME group and their siblings to perform worse than controls on most measures, suggesting that the similarity between patients and their siblings is independent of the effects of medications or subclinical EEG activity^8^.

In another study by Turkoglu et al.^9^, 22 patients with JME and 20 healthy controls were included, and all were evaluated using neuropsychological tests for executive functions and the Temperament and Character Inventory (TCI) for personality traits. Patients performed poorly on the Digit Span Test and the Stroop Color and Word Interference Test. In TCI, there was no significant difference between patients and controls. Furthermore, scores on the cooperative character dimension (social acceptance) were significantly lower in the patient group. These findings suggested that patients may have frontal lobe dysfunction, and no significant results related to personality traits were detected^9^.

Gama et al.^10^ investigated 35 patients with JME in comparison to a control group of healthy people assessed by an impulsivity scale, personality inventory, and neuropsychological tests. The group of patients presented high levels of motor impulsivity scores associated with worse control of myoclonic seizures and mild psychiatric disorders, impulsive behavior associated with lower levels of functional inhibitory control^10^.

In the study by Jackson et al.^6^, 94 children aged 8–18-years-old diagnosed with idiopathic generalized epilepsy and 71 controls underwent neuropsychological tests, and parents were interviewed about their children's academic record. It was found that children with recent-onset epilepsy presented cognitive changes in verbal memory, language, and executive functioning, and also academic problems^6^.

Epilepsy with myoclonic–atonic seizures (EMAS), or Doose syndrome, is a rare epilepsy syndrome that affects approximately 1–2% of children with epilepsy. It was described in 1970 for the first time as "Centrencephalic Myoclonic Astatic Petit Mal," by the neuropediatrician Hermann Doose. It has been described as generalized epilepsy that begins in children between 7 months and 6 years of age, often with an onset of various types of seizures^11^. Seizures can include distinct dropping attacks due to myoclonic–atonic components. Other types of seizures include absence, tonic–clonic, generalized, and tonic seizures in the most severely affected children^12,13^. EMAS is now considered to be a developmental and epileptic encephalopathy.

Only a small percentage of those patients have pathogenic variants of the *SCN1A* gene and variable outcomes from normal intellect to severe intellectual disability^14-16^. Although it is associated with several types of generalized seizures, the diagnosis is based on the presence of myoclonic–atonic seizures^17^. In total, 18% of patients have refractory seizures and intellectual disabilities^18,19^. Some children may present behavioral changes, such as hyperactivity, and others may develop refractory seizures, severe intellectual deficits, and serious behavioral problems, even if the epileptic seizures disappear^6,20^. Studies investigating cognitive and behavioral impairments in children with this type of epilepsy are even more scarce.

Ding et al.^7^ described that children with EMAS usually have normal mental and motor development before the onset. Most children start with a generalized tonic–clonic seizure (GTCS). The focal seizures can be very frequent, followed by a variety of generalized seizures, including myoclonic seizures and atypical absence. A small number of children have tonic seizures in the later stages^7^. In a case study of EMAS, the patient started with a febrile seizure at 3 years of age, after which subsequent multiple myoclonic and myoclonic–tonic seizures appeared^21^.

Hinokuma et al.^22^ investigated the genetic context and genotype–phenotype correlations of 29 patients with EMAS and found that seizures preceded epileptic or afebrile seizures in four patients, of whom two had genetic variants. Myoclonic–atonic seizures occurred at onset in four patients, of whom two had genetic variants, and during the course of the disease in three patients. Genetic variants were more commonly identified in patients with a developmental delay or intellectual disability. Attention deficit hyperactivity disorder (ADHD) and autism spectrum disorder (ASD) were less frequently observed in patients with genetic variants than in those with unknown etiology^22^.

Despite these previous findings, detailed cognitive and behavioral performance in patients with EMAS remains largely unknown. Therefore, the aim of this study was to investigate the cognitive and behavioral performance in patients with EMAS compared to a control group, including measures of adaptive behavior, intelligence quotient (IQ), episodic memory, language, attentional and executive functions, and the presence of ASD and ADHD traits. The accurate investigation of these cognitive and behavioral abilities is essential for an appropriate diagnosis and management. Furthermore, there are no parameters in the Brazilian literature regarding the performance of these patients on adaptive behavior, ASD, ADHD, and specific cognitive functions.

## METHODS

In this study, we investigated cognitive and adaptive behavior changes in children diagnosed with EMAS compared to a healthy control group. It is a cross-sectional cohort study, developed at the Department of Neurology of the University of São Paulo Medical School (Cappesq—14090, 1.412.393). A total of nine patients referred by the Child Neurology Outpatient Clinic, Epilepsy group of the Central Institute of the Clinical Hospital of the University of São Paulo Medical School and nine healthy controls recruited from the community underwent neuropsychological (see below) and adaptive behavior assessment (VINELAND II)^23^, in addition to investigating the presence of symptoms related to ADHD using the SNAP IV^24^ and ASD using the CARS^25^. Participation was voluntary; the parents or guardians were informed that it would not cause any type of harm or changes to the ongoing treatment received, and all signed an informed consent form.

The total sample consisted of nine children diagnosed with EMAS, nine controls, aged 6–12 years old, recruited during the years from 2014 to 2018. The group of patients was using epilepsy medications during the neuropsychological assessment and did not have seizures 24 h before the assessment that could cause potential damage (data can be seen in Table 1). The mean age in months of onset of the first seizure in patients with EMAS was 36.4 months (M=36.4, DP±13). The potential side effects of the medication that could interfere with performance during cognitive assessment would be drowsiness and the presence of seizures. However, patients during this period did not present with seizures or drowsiness during the assessment sessions. The healthy control group was evaluated by the same neuropsychological protocol described below.

**Table 1:** Medications administered and periodicity of seizures for each patient.

Patient	MAC	Dosage	PS	LS (days)
1	Without medication	-	No seizures for 1610 days	1,610
2	Topiramate	500 mg/day	Weekly	20
	Sodium valproate	2 g/day		
	Clobazam	40 mg/day		
3	Topiramate	300 mg/day	No seizures for 210 days	210
	Sodium valproate	25 mg/day		
	Neuleptil	3 mg/day		
4	Without medication	-	No seizures for 910 days	910
5	Valpakine	1500 mg/day	Annual	365
	Levetiracetam	875 mg/day		
	Ethosuximide	825 mg/day		
6	Topiramate	400 mg/day	Weekly	7
	Sodium valproate	1,625 mg/day		
	Levothyroxine	100 mg/day		
	Imipramine	50 mg/day		
	Quetiapine	50 mg/day		
7	Lamotrigine	150 mg/day	No seizures for 120 days	120
	Sodium valproate	500 mg/day		
8	Nitrazepam	15 mg/day	Weekly	7
	Sodium valproate	750 mg/day		
	Topiramate	400 mg/day		
9	Without medication	-	Annual	365

Abbreviations: MAC, medication administered continuously; PS, periodicity of seizures; LS, last seizure before evaluation.

Clinical interviews were carried out with parents or guardians. They answered questions regarding identification, neuropsychomotor development, treatments carried out, and the application of adaptive behavior scales, ASD, and ADHD. Patients and controls were assessed by neuropsychological tests of intelligence^26^, sustained attention^27^, verbal auditory episodic memory^28^, visuospatial episodic memory, language^29^, executive functioning, related to planning, phonological and semantic verbal fluency, inhibitory control, and visuoconstruction^30^. During the period in which the evaluation was carried out, the patients did not present drowsiness or seizures in the last month or during the investigation period.

For statistical analysis, IBM-SPSS for Windows version 22.0 software was used, and Microsoft Excel 2013 software was used for data tabulation. Results were described as mean, standard deviation, and percentile using Mann–Whitney tests and Kruskal–Wallis^31^.

## RESULTS

The group with EMAS presented a mean age of 119.2 months (SD±22), 3.7 years for education (SD±2.3), and a 36.4 onset age in months of the first seizure (SD±13). The healthy control group had a mean age of 97.1 months (SD±20.9) and 2.4 years for education (SD±1.8). The group of patients and controls did not show statistical differences in terms of age and education.

Results of the scales that assessed adaptive behavior, ASD, and ADHD can be seen in Table 2. There was no significant difference between groups, except for a tendency of the SNAP-IV scale, which assesses symptoms of inattention and hyperactivity (p=0.052) in patients with EMAS showing a higher number of symptoms.

**Table 2:** Results of attention deficit hyperactivity disorder, autism spectrum disorder, and adaptative behavior scales for patients and controls.

Variable	Comparison	Value Z	P-value
SNAP-IV			
Attention	Control vs EMAS	-1.94	0.052
Hyperactivity	Control vs EMAS	-1.70	0.089
Autism scale (CARS)	Control vs EMAS	0.78	0.433
Vineland communication	Control vs EMAS	1.36	0.173
Vineland autonomy	Control vs EMAS	0.42	0.678
Vineland socialization	Control vs EMAS	1.47	0.141
Adaptive behavior	Control vs EMAS	0.91	0.365

For the neuropsychological tests, there were significant differences between the groups (p<0.05), with poorer performance for the group of patients with EMAS. The results of the neuropsychological tests are described in Table 3. The groups differed on the estimated IQ Test (p=0.002) and RAVLT delayed recall, which assesses verbal episodic memory (p=0.008). There were also significant differences for the executive and attention domains including tests of phonological (p=0.024) and semantic verbal fluency (p=0.002), Rey's figure-copy (p=0.019), which assesses visuoconstruction, and Digit Span and Cancellation, which assess auditory and sustained attention (p<0.001).

**Table 3:** Results of comparisons between groups for intelligence and neuropsychological tests.

Variable		Group		Total (n=18)	p-value
		Control (n=9)	EMAS (n=9)		
Vocabulary Wasi	Gross average±SD	86.4±16.3	42.6±37.6	64.5±36.1	**0.019**
	Median (min; max)	94 (81.5; 97.5)	27 (13; 88)	85 (27; 94.8)	
Matrices Wasi	Gross average±SD	52.2±32.5	6±10.1	29.1±33.3	**0.003**
	Median (min; max)	50 (23; 82.5)	1 (0.5; 10)	15 (0.9; 55)	
Estimated QI	Gross average±SD	72.9±27.8	21.3±26	47.1±37.2	**0.002**
	Median (min; max)	82 (54.5; 89.5)	5 (2.5; 40.5)	40.5 (4.8; 83.3)	
RAVLT immediate	Gross average±SD	37.9±24.3	20.8±26.6	29.4±26.2	0.077
	Median (min; max)	37 (19.5; 51.5)	18 (0.1; 32)	26 (6; 40.5)	
RAVLT late	Gross average±SD	49.1±26.1	15.5±18	32.3±27.8	**0.008**
	Median (min; max)	61 (20.5; 61)	7 (0.3; 27)	27 (5.4; 61)	
RAVLT recognition	Gross average±SD	29.6±22.5	29±31.1	29.3±26.3	0.666
	Median (min; max)	32 (11.5; 39.5)	18 (0.5; 58)	25 (4.3; 47.8)	
Rey's figure (late)	Gross average±SD	16.9±19	6.1±8.5	11.5±15.3	0.113
	Median (min; max)	6 (5; 32)	1 (0.1; 11)	5.5 (0.9; 14.5)	
Boston naming	Gross average±SD	58±39;8	25.6±39.6	41.8±42	0.063
	Median (min; max)	58 (14.5; 97)	2 (0.1; 58.5)	21.5 (1; 91.5)	
FAS	Gross average±SD	27.6±31.1	6.3±15.4	17±26.2	**0.024**
	Median (min; max)	7 (1; 52.5)	0.5 (0.3; 3.5)	21.5 (1; 91.5)	
Semantic (animal)	Gross average±SD	33.8±20.4	6±7.4	19.9±20.7	**0.002**
	Median (min; max)	34 (14.5; 49)	2 (1; 12.5)	12.5 (1.8; 36)	
Rey's figure (copy)	Gross average±SD	31.4±29.3	11.1±21.9	21.3±27.2	**0.019**
	Median (min; max)	16 (11; 59.5)	2 (0.1; 11)	11 (1.6; 32.5)	
Cancellation	Gross average±SD	9±10.2	2±3	5.5±8.1	**0.031**
	Median (min; max)	5 (1; 20.5)	0.5 (0.1; 3.5)	1.5 (0.5; 6)	
Digits	Gross average±SD	56.1±31.6	3.9±6.9	30±34.8	**<0.001**
	Median (min; max)	63 (31; 91)	0.5 (0.1; 9)	16 (0.4; 63)	

Mann–Whitney test.

On the other hand, patients with EMAS showed preserved results on tests of immediate verbal episodic memory recall (p=0.077), verbal episodic recognition memory (p=0.666), Rey's figure delayed recall, which measures visuospatial episodic memory (p=0.113), and language in terms of naming (p=0.063).

## DISCUSSION

The current study aimed to evaluate the cognitive and behavioral performance of patients diagnosed with EMAS compared to a healthy control group using tests and scales of attention, episodic memory, language, executive functioning, adaptive behavior, and symptoms of ADHD and ASD. The two groups were well matched and did not differ statistically regarding age and years of education.

A tendency toward significant difference was found between the two groups in the results of the ADHD scale, with the patient group showing a higher number of symptoms. These results were similar to those found in a previous study, which evaluated a group of patients with EMAS and found increased levels of impulsivity that were correlated with the presence of myoclonic seizures^10^.

There were no significant symptoms of ASD in the EMAS group or controls. This result is consistent with previous studies showing normal development prior to seizure onset.

In terms of cognitive functioning, the EMAS group showed worse performance on tests of intelligence with difficulties in generating meaning of words (vocabulary subtest), ability to reason from visual stimuli, fluidity of intelligence, and logical reasoning (matrix subtest). Previous studies showed variable prognosis outcomes for EMAS patients, ranging from normal cognition to moderate intellectual disability^17^.

In addition, the EMAS group showed impairment in relation to the control group in tests of verbal auditory episodic memory delayed recall. On the other hand, they showed normal performance on the recognition of these words, suggesting that they were able to store them despite the difficulties in spontaneously retrieving those words. This could be associated with the attentional and executive demands during the spontaneous delayed free recall. In fact, they showed significant impairment in tests of attention and executive functioning, including those of phonological and semantic verbal fluency, use of strategies, inhibitory capacity, planning, and visuoconstructive abilities. Performance on auditory and sustained attention was also impaired. These findings are in line with the results from the study by Jackson et al.^6^, which found cognitive changes related to intelligence, verbal learning, memory, attention, and multiple cognitive functions in this population.

It should be noted that the EMAS group was free of seizures during the time of the present study, and as shown in previous studies, the control of seizures is directly related to the improvement of cognitive performance, as described in the study by Gama et al.^10^.

Although these children were using continuous medications, which can cause cognitive changes and be a limitation, these medications were administered in therapeutic doses. In addition, these patients did not present any occurrence of seizures 24 h before the neuropsychological assessment.

In conclusion, the current findings provide evidence of the presence of cognitive impairments in executive and attention domains, which could be accentuating or producing the verbal episodic memory deficits found in the EMAS group. On the other hand, the finding of the presence of some ADHD symptoms in this group could also explain part of the cognitive deficits they presented. Future studies could include the investigation of ADHD comorbidity in addition to cognitive and behavioral tests to elucidate this issue further.

## Data Availability

The data will be available upon request.
